# Targeted Memory Reactivation During REM Sleep in Patients With Social Anxiety Disorder

**DOI:** 10.3389/fpsyt.2022.904704

**Published:** 2022-06-20

**Authors:** Francesca Borghese, Pauline Henckaerts, Fanny Guy, Coral Perez Mayo, Sylvain Delplanque, Sophie Schwartz, Lampros Perogamvros

**Affiliations:** ^1^Department of Basic Neurosciences, Faculty of Medicine, University of Geneva, Geneva, Switzerland; ^2^Swiss Center for Affective Sciences, University of Geneva, Geneva, Switzerland; ^3^Human Neuroscience Platform, Fondation Campus Biotech Geneva, Geneva, Switzerland; ^4^Center for Sleep Medicine, Geneva University Hospitals, Geneva, Switzerland; ^5^Department of Psychiatry, Geneva University Hospitals, Geneva, Switzerland

**Keywords:** sleep, REM sleep, dreaming, social anxiety, targeted memory reactivation, exposure therapy

## Abstract

**Background:**

Social anxiety disorder (SAD) is characterized by a significant amount of fear when confronted to social situations. Exposure therapy, which is based on fear extinction, does not often lead to full remission. Here, based on evidence showing that rapid eye movement (REM) sleep promotes the consolidation of extinction memory, we used targeted memory reactivation (TMR) during REM sleep to enhance extinction learning in SAD.

**Methods:**

Forty-eight subjects with SAD were randomly assigned to two groups: control or TMR group. All patients had two successive exposure therapy sessions in a virtual reality (VR) environment, where they were asked to give a public talk in front of a virtual jury. At the end of each session, and only in the TMR group (*N* = 24), a sound was paired to the positive feedback phase of therapy (i.e., approval of their performance), which represented the memory to be strengthened during REM sleep. All participants slept at home with a wearable headband device which automatically identified sleep stages and administered the sound during REM sleep. Participants' anxiety level was assessed using measures of parasympathetic (root mean square of successive differences between normal heartbeats, RMSSD) and sympathetic (non-specific skin conductance responses, ns-SCRs) activity, and subjective measures (Subjective Units of Distress Scale, SUDS), during the preparation phase of their talks before (T1) and after (T2) one full-night's sleep and after 1 week at home (T3). Participants also filled in a dream diary.

**Results:**

We observed an effect of time on subjective measures of anxiety (SUDS). We did not find any difference in the anxiety levels of the two groups after 1 week of TMR at home. Importantly, the longer the total duration of REM sleep and the more stimulations the TMR group had at home, the less anxious (increased RMSSD) these participants were. Finally, fear in dreams correlated positively with ns-SCRs and SUDS at T3 in the TMR group.

**Conclusion:**

TMR during REM sleep did not significantly modulate the beneficial effect of therapy on subjective anxiety. Yet, our results support that REM sleep can contribute to extinction processes and substantiate strong links between emotions in dreams and waking stress levels in these patients.

## Introduction

Social anxiety disorder (SAD) is characterized by an exaggerated and persistent amount of fear when confronted with social situations (1), which can lead individuals with SAD to either avoid such situations or endure them with significant discomfort (1, 2). It is a chronic disorder with a lifetime prevalence rate of 13% and an early onset in adolescence (3, 4). Studies looking into the mechanisms underlying anxiety disorders converge to suggest that these disorders are characterized by dysfunctional fear extinction (5).

Fear conditioning is a form of associative learning between a neutral stimulus (conditioned stimulus CS) and an innately aversive stimulus (unconditioned stimulus US), after repeated pairings of the CS and the US. After fear conditioning, presentation of the CS alone triggers an emotional response (conditioned response, CR) (6, 7). Fear extinction learning (or inhibitory learning) is a process during which the conditioned fear response decreases or is inhibited when there is a repeated presentation of the CS in the absence of the US. Existing data support that extinction induces the learning of a new association “CS-noUS,” in which the CS no longer predicts the US (5, 8). Inhibitory learning is central to extinction and deficit in this process could contribute to the development of anxiety disorders (6, 9).

Exposure therapy is a treatment based on extinction learning mechanisms and involves the gradual and repeated exposure to the feared stimuli in the absence of the negative outcome (5). It is a popular and efficient treatment of SAD and other anxiety disorders such as generalized anxiety disorder (GAD) and specific phobias (10). However, while there is a consensus in the field of psychotherapy regarding the efficiency of exposure therapy to treat SAD, patients are reluctant to seek treatment, as they can be hesitant to engage in social interactions. The use of virtual reality (VR) allows a better control of exposure conditions and the ability to stop or take breaks if the patient is overwhelmed (11). A meta-analysis conducted by Carl et al. (12) indicated that virtual exposure and *in vivo* exposure showed similar efficacy in the treatment of anxiety disorders (including SAD), both leading to reduction of anxiety symptoms. However, exposure is not a foolproof solution to treat anxiety disorders, as its efficacy is not always significant in the long run. Many patients experience a return of fear at the end of the exposure therapy, with rates up to 62% (5). Therefore, there is an emerging need to find ways to enhance the therapeutic outcome of this therapy.

In order to enhance extinction learning, before, during or after exposure therapy, several methods have been used, such as administration of cortisol (13) or stimulation of medial prefrontal cortex (mPFC) with repetitive transcranial magnetic stimulation (14). A simple positive reinforcement or feedback (e.g., positive compliments) regarding the patient's performance, which represents extinction-related violation of expectancy (5), was also found to reduce social anxiety (15), and is an integral part of self-focused exposure therapy (16). Other protocols have used periods of sleep (naps or full night's sleep) to reinforce the consolidation of extinction learning after exposure therapy for anxiety disorders, such as spider phobia (17, 18). A preliminary study recently demonstrated that naps after exposure therapy for SAD lowered sympathetic responses (as measured by skin conductance response and cortisol levels) during anticipation of a social challenge at a trend level (19). In their conclusion, these authors suggested that the lack of significance could potentially be due to the fact that post-exposure sleep was not long enough for the participants to experience rapid eye movement (REM) sleep, and that REM sleep could play an important role in the consolidation of extinction learning.

Indeed, accumulating evidence shows that REM sleep may represent a permissive condition for the processing of extinction memory. Healthy participants who had REM sleep after extinction learning exhibited greater extinction recall, accompanied by stronger activation of the ventromedial prefrontal cortex (vmPFC) in response to the extinguished stimulus (20, 21). Moreover, a greater retention of extinction learning was associated with REM percent in an intervening overnight sleep (22), while a subsequent study elegantly demonstrated that REM sleep (but not slow wave sleep-SWS or wakefulness) causes successful consolidation of extinction memory (23). Other studies also showed that REM sleep helps to decrease the experienced arousal or affective tonus associated with emotional events, thus leading to higher familiarity and habituation to emotionally negative stimuli (emotional depotentiation) (24, 25). Recent neuroimaging data also indicated that negative emotions in dreams, and specifically fear, may contribute to (or reflect) emotional regulation processes during sleep and yield better adapted responses to aversive stimuli during waking life (26).

Importantly, most of the previous studies have been restricted to analyzing the effects of a single night of sleep [or using a split-night design (23)] on fear conditioning and extinction. A recent study (27), showed that baseline REM sleep duration measured over several days (mean, 7.88 days; range, 5–13 days) can predict subsequent reduced fear-related activity in the amygdala, hippocampus, and vmPFC, supporting the enhancing role of this sleep stage not only in extinction, but in reducing fear conditioning too. This effect was present but weaker when markers of fear acquisition were related to a single night of measurement. Such studies (27, 28) stress the importance of assessing REM sleep over several nights to better predict the future level of conditioning (i.e., there is a trait-level rather than state-level effect of sleep on fear conditioning and emotional reactivity).

Memory reactivation during sleep can be induced or intensified with targeted memory reactivation (TMR). This method consists in associating a sensory cue with a learning experience, and subsequently presenting this cue to increase the likelihood that the memory of this experience is reactivated. Thus, presenting the cue during sleep will trigger a neuronal replay of the associated memory, which will strengthen memory consolidation (29). Rasch et al. (30) showed that presenting during sleep an odor, which was previously associated with a learning phase (location of objects), improved the retention of the learned information, as shown by a superior memory performance when tested post-sleep. Such a reactivation during sleep can take place when using odor cues, but also auditory cues (31, 32). Studies have demonstrated that such cued memory reactivation can improve the consolidation of declarative and procedural memories to levels up to 35% as compared to wakefulness (31, 33, 34). While the benefits of applying TMR during non-REM (NREM) sleep have been established across many studies, results are more inconsistent when cueing occurs during REM sleep, due probably to the predominance of nap studies (containing no or only short periods of REM sleep) (34). TMR during REM sleep enhanced memory (35, 36), including associative emotional memory and generalization (37) [contrary to SWS; Ashton, Cairney (38)], while it increased positive valence of negative stimuli (39, 40) and reduced emotional arousal (41). Therefore, using TMR during REM sleep could be an efficient method to enhance extinction memory consolidation and improve inhibition learning, which is lacking in individuals presenting anxiety disorders.

Lastly, a dominant theory suggests that memories are initially encoded into a fast-learning store (i.e., the hippocampus) and are gradually transformed into a long-term storage (i.e., the cortex) during consolidation (42, 43). In order to avoid the return of fear after extinction (44) and to permanently consolidate the formation of the new (i.e., initially labile) safety memory during sleep, TMR during REM sleep may have to be repeated over several successive nights (at least 1 week), the time needed to make the extinction memory hippocampus-independent (45).

The main goal of this study was to investigate whether TMR during REM sleep over several consecutive nights may enhance exposure therapy in SAD. Even though REM sleep and dreaming appear to have an important role in extinction learning and emotional depotentiation, to our knowledge, no study to date used TMR during REM sleep in the context of treating anxiety disorders. In our study, participants with SAD took part in virtual reality exposure sessions during which they were asked to perform public presentations. Anxiety levels were assessed at different time points with subjective and physiological measures. Specifically, the anticipatory phase of their performance, being particularly stressful (46–48), was chosen as a critical period for stress measurement. Following the public presentations in VR, participants received positive feedback of their performance (16), which served as an extinction period of the therapy. Indeed, as there are no negative consequences such as negative judgment from the jury following the feared situation, this period represents a CS-noUS association reminiscent of extinction learning (5). During this period, participants in the experimental group (TMR group) were exposed to an auditory cue, while those in the control group were not. In the frame of the TMR technique, this allows for an association between the sound and the extinction memory. During eight nights following the first virtual exposure session, participants from both groups received the auditory cue selectively during REM sleep at home.

The primary hypothesis of this study was that (a) participants in the TMR group will show reduced intensity of social anxiety compared to participants in the control group, based on subjective reports and physiological measures, after eight nights of stimulations during REM sleep. Secondary hypotheses included the following: (b) the aforementioned effect could be already observable after one night of sound presentation during REM sleep; (c) participants in the TMR group will experience generalization of extinction compared to participants in the control group, after eight nights of sound presentation during REM sleep; and (d) the number of stimulations in the TMR group and/or REM sleep duration will have a beneficial effect on stress, respectively due to increased TMR-related events and the proposed role of REM sleep in extinction learning and emotional depotentiation. Considering recent evidence on the links between dreams and emotional processing in wakefulness (26), we also hypothesized that: (e) fear in dreams should positively correlate with reduced stress in wakefulness.

## Methods

### Participants

Participants were recruited through flyers and advertisements on social media. Inclusion criteria were: being aged between 16 and 40 years old, having SAD according to the Diagnostic and Statistical Manual of Mental Disorder, 5th edition (49), not being under treatment for social anxiety, and not presenting other mental disorders or sleep disorders. Patients with symptoms of obstructive sleep apnea syndrome, restless legs syndrome, insomnia disorder, or under anxiolytics, antipsychotic or antidepressant medication were excluded. Initially, 51 participants were recruited. Three of them were not included in the final analyses because they withdrew from the study. The final sample of participants was composed of 48 participants (32 females and 16 males) with SAD, as assessed by an interview with a certified psychologist. Participants gave their written consent to take part in this study and received a participant fee. The study was approved by the Ethical Committee of the Canton of Geneva, Switzerland (“*Commission Cantonale d'Ethique de la Recherche sur l'être humain*”).

### Procedure

**Figure 1:**
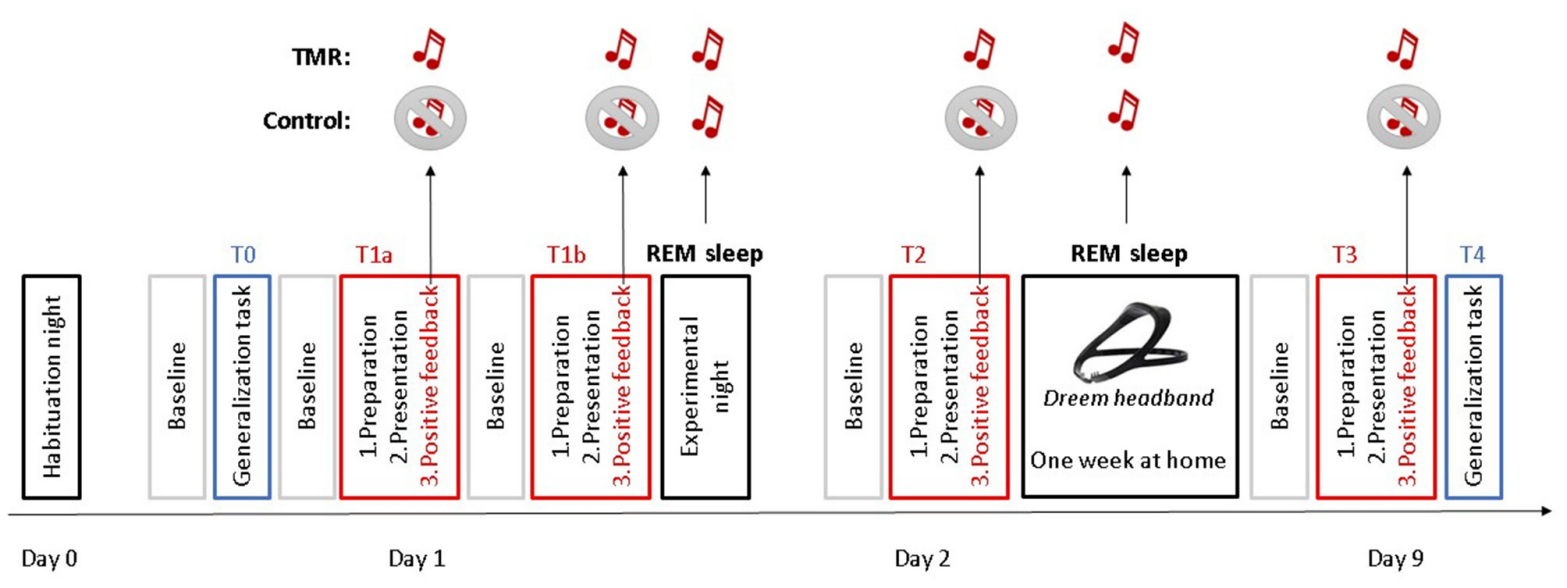
Study design. Participants underwent a habituation night on Day 0. The following day (Day 1), they had one VR session of generalization task (T0) and two VR sessions of self-focused exposure therapy (T1a and T1b), before the experimental night. On Day 2, participants underwent another VR session of exposure therapy (T2). Then, they spent 1 week at home with a headband sleep device. On day 9, participants came for one last VR session of exposure therapy (T3) and one VR session of generalization task (T4). During the positive feedback phases of the VR, a sound was administered to the TMR group, while no sound was administered to the control group. Both groups were administered the sound during their REM sleep at the experimental night and during the nights at home with a sleep headband.

Forty-eight SAD patients were randomly assigned into two groups (TMR group and control group, Figure 1). This randomization took place on Day 1 (T1). Patients of the TMR group received a neutral auditory stimulus [i.e., a 1-second piano chord (C69)], which was associated with the positive feedback phase of exposure therapy. The aim was to consolidate this new associative memory during REM sleep through TMR. Therefore, two positive feedback phases took place on T1: T1a and T1b. The only purpose of the T1b timepoint was to further reinforce the association between the sound and the positive feedback period before the first experimental night, and it was therefore not used in the statistical analyses. After this session, all participants had a full night of polysomnography (PSG). During sleep, patients of both groups received the sound during REM sleep with a wireless sleep headband. On Day 2, the participants underwent another VR session of exposure therapy (T2). Then, they spent 1 week at home with the headband device administrating the sound during REM sleep. On day 9, participants came for one last VR session of exposure therapy (T3). Social anxiety for a public talk was measured during the preparation of the talk at six different time points: before (T1a) and after (T1b) the first session of VR exposure therapy, after one night of sleep with TMR (T2), and after 1 week of TMR at home (T3). In order to test for generalization of extinction, we tested anxiety for another context (being approached by virtual characters) at time points T0 and T4. Throughout all phases of the VR sessions (baseline, preparation, presentation, and positive feedback) (see Section ‘Structure of Each VR Exposure Session' for details), anxiety was measured at the subjective level (Subjective Units of Distress Scale) and physiological level, including heart rate variability and electrodermal activity.

### Materials

#### VR Environment

Participants were immersed in a virtual environment, with an Oculus Rift Headset (Meta Quest, Irvine, California, United States). The virtual environment was designed on Unity 3D (Unity Software Inc., San Francisco, United States) by the Virtual Reality and Robotics facility of the Human Neuroscience Platform, Fondation Campus Biotech Geneva. The virtual environment consisted of a theater stage and an audience space. On the stage, there was a microphone stand in front of which the participant made his/her presentations. From the stage, the participants could see the audience space, where there was a table (with a timer on it), two virtual jury members (one male and one female) and a virtual audience of about 20 members. Electrodes for the recording of electrodermal activity and electrocardiogram signals were placed on the participant before he/she entered the booth, where the VR sessions took place. The participant could communicate and hear the instructions from the experimenter through the headphones and microphone included in the VR headset.

#### Structure of Each VR Exposure Session

All the VR sessions of the main task (T1a, T1b, T2, and T3) in this experiment have the same structure (see Supplementary Material 1 for detailed description). They started with a 3-min baseline phase during which participants had to stay seated, relaxed and get used to the environment (baseline phase). The goal of this phase was to allow any distress the participant might feel to dissipate. After this phase, they were allowed 5 min to mentally prepare a short speech on a given topic (preparation phase), which they would present afterwards for 5 min in a virtual theater room with a two-person jury and a public in the background (presentation phase). At the end of their performance, a virtual feedback was given to them: first, a standard positive feedback from the virtual audience and jury (0.5 min), and then the experimenter gave an individualized positive feedback (3 min). During this phase (positive feedback phase), a sound was administered every 10 s in the TMR group, while no such sound was administered in the control group.

#### Sleep Headband Dreem^®^

The Dreem^®^ headband is a wireless sleep headband designed by the company Dreem (Dreem SAS, Paris, https://dreem.com/). It is composed of fabric and foam and can be adjusted with an elastic band behind the head. The device records and analyses physiological data: (1) brain activity *via* EEG dry electrodes (derivations: FpZ-O1, FpZ-O2, FpZ-F7, F8-F7, F7-O1, F8-O2, FpZ-F8; 250 Hz with a 0.4–35 Hz bandpass filter), (2) movements, (3) sleep position, and (4) heart rate. The EEG electrodes are placed at the front and back of the device. Sound can be delivered *via* bone-conduction transducers integrated in the headband. The headband monitors sleep and can detect different sleep stages through a reliable algorithm (50). In our experiment, whenever REM sleep was detected for more than 5 min, the sound was delivered to the participants every 10 s. These auditory stimulations were interrupted whenever a new sleep stage was detected (after which they restarted after 5 min of REM), after detection of a movement (90 s of interruption), after detection of an alpha wave (45 s of interruption), after detection of a blink (10 s of interruption), and after bad quality signal (4 s of interruption). The headband was connected to a smartphone application, which provided a summary on sleep quality, and allowed each participant to choose the volume of the sound. Remote access to the raw sleep data and stimulations per night was possible, with daily controls ensuring patient compliance to the protocol.

#### Questionnaires

As part of the recruitment, participants filled several online questionnaires used to verify inclusion criteria: general socio-demographic and medical questionnaires, the Liebowitz Social Anxiety Scale (LSAS) (51), the Pittsburgh Sleep Quality Index (PSQI) (52), the Beck Anxiety Inventory (BAI) (53), the Beck Depression Inventory II (BDI II) (54), and the Insomnia Severity Index (ISI) (55). During initial assessment, a certified psychologist used the Mini-International Neuropsychiatric Interview (MINI) module on social anxiety disorder (56), a short structured diagnostic interview instrument, based on the DSM-5 criteria.

The LSAS was administered to assess the presence and degree of social anxiety, while the MINI was used in order to establish a SAD diagnosis according to DSM-5. The PSQI and ISI were administered in order to detect and exclude participants with sleeping issues, as the latter could interfere with the study. Finally, we also used the BAI and BDI-II to detect anxious and depressive symptoms, respectively. The internal consistency rates of these questionnaires, as assessed in this study, were calculated with Cronbach's alpha and are considered good/excellent for LSAS (α = 0.81), BAI (α = 0.94), BDI-II (α = 0.82), and acceptable for PSQI (α = 0.71) and ISI (α = 0.77).

#### Dream Diary, Sleep Agenda, Psychomotor Vigilance Task

During 2 weeks (starting from 1 week before the first VR session and finishing at the end of the protocol), participants filled out a dream diary and a sleep agenda. The dream diary was filled in every morning upon awakening [see also (26)]. Upon awakening, participants were asked to report whether they had a dream with or without recall or no dream at all, during the immediately preceding night. Whenever they reported having a dream with recall, they were asked to answer additional categorical questions related to the length, clarity, perceptual features, and emotionality of the dream (i.e., whether they experienced fear, anger, frustration, sadness, embarrassment, confusion, disgust, and joy). Specifically regarding the emotions, participants had to choose whether they were feeling a particular emotion in a dichotomous way, by stating if this emotion was present or not in their dreams. Finally, they were asked to freely describe the dreams they had experienced during their sleep. In the present study, we focused on the closed-ended questions about the emotions experienced in the dream.

Before each VR session, participants also performed a psychomotor vigilance task (PVT), which is a sustained-attention, reaction-timed task (57). Lasting for 5 min, this task consisted in pressing on a computer key as fast as possible, as soon as a millisecond timer appeared on the screen after the disappearance of a fixation cross. This allowed us to ensure that the participants in the two groups did not differ in their general vigilance state during the VR sessions. Additionally, after the two nights at the lab, and after 1 week of sleeping at home, participants filled out post-sleep the St. Mary's Hospital questionnaire (58) to evaluate the quality of their sleep.

### Measurements

#### Measures of Anxiety

During the VR tasks, we assessed stress levels with two physiological measures: heart rate variability (HRV) (59–61) and electrodermal activity (EDA) (62, 63), which provide estimates of parasympathetic and sympathetic nervous system activity, respectively. We have also used a subjective measure of anxiety, the Subjective Units of Distress Scale (SUDS) (64).

HRV was measured using three ECG electrodes (including one ground) placed below the rib (left), under the clavicle (right) and on the hip (right) for the ground. For our study, we were interested in the root mean square of successive differences between normal heartbeats (RMSSD, in ms) for each VR session phase. The RMSSD is used to estimate the vagally mediated changes in heart rate variability (HRV) (65) and presents the advantage of closely representing parasympathetic activity (66), and being relatively free of respiratory influences compared to other variables calculated from HRV (67). Lower levels of RMSSD have been associated with higher anxiety in several anxiety disorders, including SAD (66, 68).

EDA is a term used to define autonomic changes in the electrical properties of the skin and is used as an objective index of emotional stress (62). It was measured with disposable adhesive sensors on the distal phalanges of the index and middle finger of the non-dominant hand of participants. EDA was analyzed to obtain skin conductance responses (SCRs). For our study, we were interested in the non-specific SCRs (ns-SCRs), which are spontaneous, phasic increases in EDA that are not associated with any specific stimuli (62). An increased frequency of ns-SCRs is considered a biomarker of high arousal in situations of stress, emotional reactivity and anticipatory anxiety (46, 62, 69).

The adapted Subjective Units of Distress Scale, SUDS (64, 70) was used during the Virtual Reality (VR) sessions to measure the intensity of distress of people suffering from social anxiety. It consists in a single question scale, in which the subject rates on a scale from 0 to 10 the level of distress that they were feeling at a specific moment. The SUDS was given after each positive feedback and preparation phase, after T0, as well as before the preparations T2 and T3.

Both ns-SCRs and RMSSD were recorded using the Biopac MP160 System (Biopac Systems Inc., Goleta, CA, 2013), and the software AcqKnowledge v.5.0. Details of data analysis of ns-SCRs and RMSSD are provided in Supplementary Material 2. The internal consistency rates of these measures in this study were calculated with Cronbach's alpha and are considered excellent for RMSSD (α = 0.92) and good for SUDS (α = 0.88) and ns-SCRs (α = 0.84).

In this study, the primary psychophysiological outcome measure was the RMSSD, during the preparation phase of exposure therapy (67). The primary subjective measure of distress was the SUDS collected immediately after the end of the preparation phase and before the oral presentation in the VR environment. The secondary outcome measure was the ns-SCRs (46) during the preparation phase of exposure therapy. The anticipatory phase of social performance (e.g., preparation of public speaking) was chosen as the main phase to study social anxiety, as it has been shown to be particularly stressful (71). We have originally selected the RMSSD as the primary physiological measure, while ns-SCRs as the secondary outcome measure, as it has been shown that heart rate measures may be more sensitive in measuring social anxiety than electrodermal activity (66, 68, 72).

Other exploratory variables included the change of fear in dreams (average of fear during the 2nd week with stimulations minus the 1st week without stimulations) (see also Section ‘Emotional Dream Content’).

#### Polysomnography

To assess the sleep structure of the participants we recorded polysomnography (PSG) for two nights at the sleep laboratory (one habituation night without auditory stimulations, one experimental night with auditory stimulations). There were six electrodes (F3, F4, C3, C4, O1, and O2) placed on the head using the 10–20 system. We also put two electrodes to measure the eye movements (EOG1 and EOG2) and three electrodes to measure muscle tone (EMG1, EMG2, and EMG3). We placed the references on the mastoids and the ground was placed on the cheekbone. We also placed two electrodes to measure the heart rate (ECG). The setup and sleep scoring were done according to the AASM manual guidelines (73).

#### Sample Size Consideration

Based on a previous study (74) on the difference of SAD vs. control group for HRV with an effect size of *d* = 0.77, 44 patients (22 per arm) would be required to have an 80% chance of detecting this difference between patients with TMR vs. those in the control group. Besides, based on a study (37) on the effect of associated vs. non-associated sound during REM sleep on associative memory with a large effect size (*g* = 1.01), this sample size would be sufficient for the aforementioned detection.

### Statistical Analysis

#### Levels of Anxiety

These analyses were done on 46 participants (two participants from the initial sample were excluded due to unusable data). Data were entered into a multilevel regression model, with either RMSSD levels or SUDS scores, as dependent variables, and time and group as (interacting) independent variables. The latter represented the fixed effects of the multilevel model, while random effects were represented by a random intercept for subjects [Y ~ group^*^time + (1|ID)]. The random intercept accounted for correlation between repeated measures, by assuming baseline differences between subjects in the average DV. A multilevel regression was chosen for these data, due to its ability to handle (a) missing data in the time variable, (b) time-varying covariates, and (c) continuous within-subject covariates. Once the model was fitted, we performed a Type II ANOVA breakdown of fixed effects using *F*-tests, starting with the interaction test of Time × Group, followed by main effects tests for Time and Group separately. Multiple testing correction for eventual follow-up pairwise comparisons was done using the Bonferroni method. As there were 3 time points (T1a, T2, and T3), a threshold of 0.0167 (= 0.05/3) for determining significance was used. Degrees of freedom for all *F*- and *t*-tests were adjusted for the random effects structure using Satterthwaite's method (75) yielding fractional degrees of freedom. Multilevel analyses were conducted with the R statistical language, version 1.2.5019 (RStudio Team, Boston, MA, 2019), using the packages “lme4” for model estimation (76) and “lmerTest” for inferential tests (77). The secondary psychophysiological outcome measure, i.e., ns-SCRs, underwent the same analysis structure.

#### Sleep Variables

Data on several sleep variables (e.g., duration of sleep stages, total sleep time, number of auditory stimulations, and volume of sound) were delivered directly from the automatic algorithm of the Dreem headband (50) or were collected after manual scoring of the two PSGs. A correlational analysis was done between the absolute REM duration on average during 1 week at home and the stress variables (RMSSD, SUDS, and ns-SCRs) at T3 for the two groups separately. The same analysis was done with the number of auditory stimulations on average during 1 week. Before the correlations were performed, a normality test was done on the different variables that were used, to use the appropriate test.

We would like to note that we applied a purely marginal (i.e., without adjusting for covariates), model-free analysis of the relation between REM sleep and stress variables after several days of stimulation (T3). The correlations for the other sleep stages and at T2 were conducted only after the hypothesis-driven ones i.e., the relation between REM sleep and stress variables at T3 had been performed, and only for reasons of completeness and transparency.

#### Emotional Dream Content

Among all emotions reported in the dreams (see Section Materials), we focused on fear (see Introduction section), which was assessed as follows. We first assessed the presence of fear in dreams by checking whether the participants had reported having the emotion of fear in their dream or not. We then calculated the proportion of dreams containing fear in the week without the stimulations and during the week with stimulations. More specifically, we took the number of nights where this emotion was reported as being present divided by the number of nights where participants reported that they had a dream with recall. This gave us a proportion of this emotion for each participant, with one value for the week before stimulations and one value for the week with stimulations.

A correlational analysis was done between the change of fear in dreams (average of fear during the 2nd week with stimulations minus the 1st week without stimulations) and the primary outcome measures (RMSSD, SUDS) and secondary outcome measure (ns-SCRs) at T3 for the two groups separately.

## Results

Recruitment took place from July 2020 to June 2021. Supplementary Figure 1 provides a flow diagram of study participants.

### Baseline Characteristics

There were no differences between the two groups in age, social anxiety (assessed by the LSAS), sleep quality (assessed by the PSQI), anxiety (assessed by the BAI-II), depression (assessed by the BDI-II), severity of insomnia (assessed by the ISI), and vigilance at T1 (assessed by PVT), as indicated in Table 1.

**Table 1:** Means, standard deviations, and comparison between the control and TMR group, of the age and the initial scores at the Liebowitz Social Anxiety Scale (LSAS), the Pittsburgh Sleep Quality Index (PSQI), the Beck Anxiety Inventory (BAI), the Beck Depression Inventory II (BDI II), the Insomnia Severity Index (ISI), and the psychomotor vigilance task (PVT) preceding the first VR session.

	**TMR group (*N* = 24)**	**Control group (*N* = 24)**	***t* (df)**	***p***
Age	24.7 ± 5.78	24.12 ± 3.97	0.4 (40, 74)	0.68
Liebowitz	101.08 ± 12.1	100.29 ± 11.95	0.22 (45, 99)	0.82
PSQI	3.20 ± 1.28	3.08 ± 1.24	0.34 (45, 96)	0.73
BDI	7 ± 6.06	6.62 ± 5.41	0.22 (45, 43)	0.82
BAI	26.5 ± 14.85	21.16 ± 13.83	1.28 (45, 76)	0.2
ISI	3.83 ± 2.42	3.12 ± 2.32	1.03 (45, 92)	0.3
PVT	269.62 ± 36.03	263.83 ± 22.79	0.66 (38, 87)	0.5

Detailed results on the comparison between stress levels of the preparation, baseline and feedback phases of the VR sessions are reported in Supplementary Material 3, while comparison of several sleep measures across time and groups are reported in Supplementary Table 1.

### Effects of TMR on Social Anxiety (T1a, T2, and T3)

#### RMSSD

No significant group × time interaction was shown [*F*_(2,76.848)_ = 0.7393, *p* = 0.481], nor a main effect of time [*F*_(2,76.799)_ = 1.0190, *p* = 0.366]. Results showed a significant main effect of group [*F*_(1,41.035)_ = 4.1536, *p* = 0.048].

#### SUDS

Analysis on SUDS scores showed no significant group × time interaction [*F*_(2,88)_ = 0.0142, *p* = 0.986] and no main effect of group [*F*_(1,44)_ = 0.3382, *p* = 0.564]. There was a significant main effect of time [*F*_(2,88)_ = 30.0611, *p* < 0.001].

#### ns-SCRs

No significant group × time interaction was shown [*F*_(2,71.174)_ = 0.3229, *p* = 0.725], neither a main effect of group [*F*_(1,40.772)_ = 0.1041, *p* = 0.749] nor a main effect of time [*F*_(2,71.120)_ = 0.0691, *p* = 0.933].

**Figure 2:**
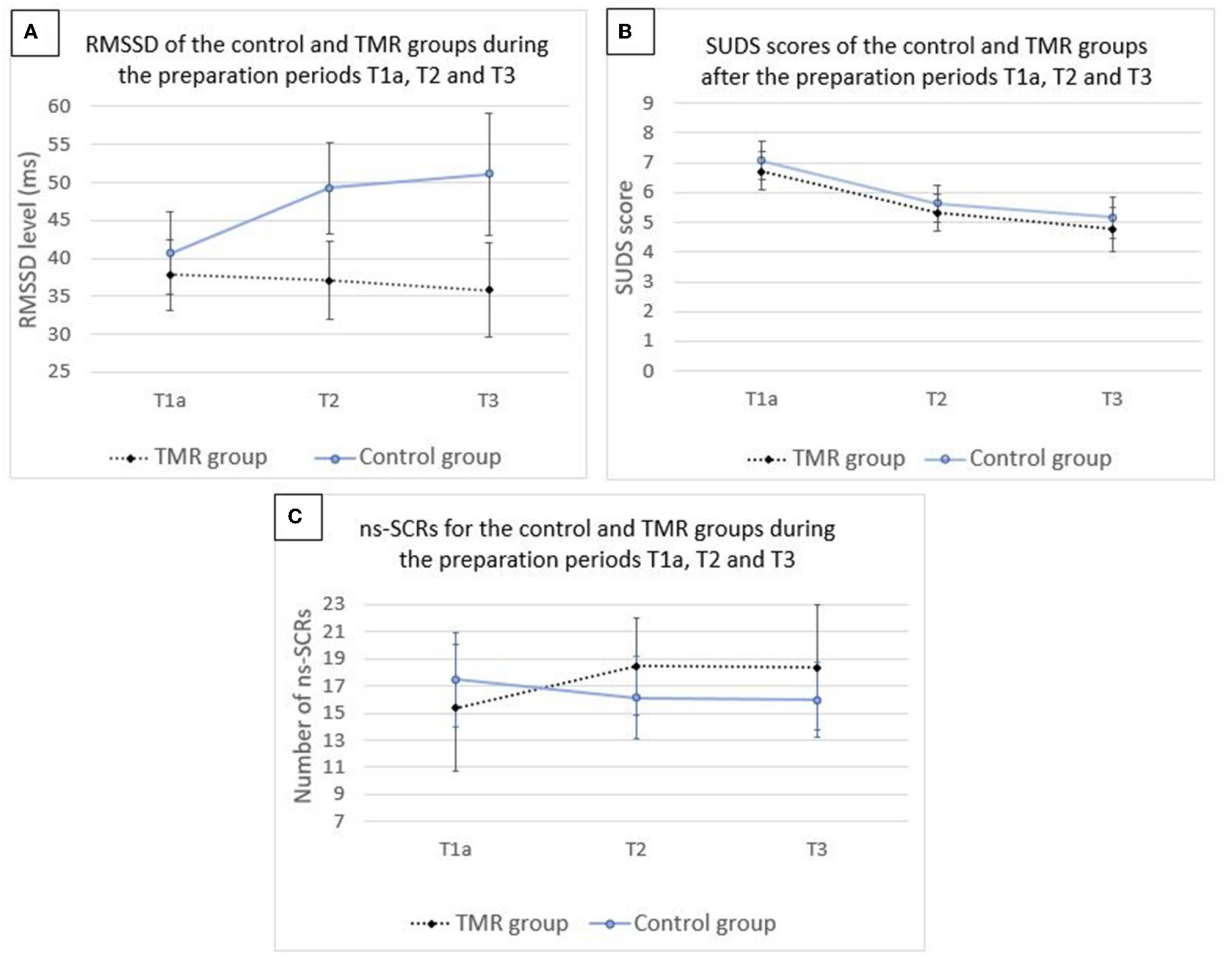
Mean values for the level of RMSSD **(A)**, score of SUDS **(B)**, and number of ns-SCRs **(C)** for the control and TMR groups during the preparation periods T1a, T2, and T3. *N* = 46. Error bars represent 95% CI.

The results are illustrated in Figure 2 and the means and standard deviations per group are reported in Table 2. Fixed effects estimates are reported in the Supplementary Table 2.

**Table 2:** Means and standard deviations of the TMR and control groups on RMSSD (ms) levels, SUDS score, and ns-SCRs (number of events) during the T1a, T2, and T3 preparation periods.

		**TMR group**	**Control group**
		***N* = 22**	***N* = 24**
T1a	RMSSD	37.79 (15.93)	40.69 (18.54)
	SUDS	6.73 (2.19)	7.08 (2.16)
	ns-SCRs	16.15 (16.03)	17.44 (12.11)
T2	RMSSD	37.14 (17.96)	49.26 (20.76)
	SUDS	5.32 (2.19)	5.625 (2.14)
	ns-SCRs	18.42 (12.25)	16.12 (10.55)
T3	RMSSD	35.83 (21.30)	51.11 (27.69)
	SUDS	4.77 (2.59)	5.16 (2.43)
	ns-SCRs	18.35 (15.93)	15.95 (9.67)

*N = 46*.

### Effect of Long-Term TMR on Generalization of Anxiety (T0 and T4)

#### RMSSD

Regarding RMSSD levels, results showed no group × time interaction [*F*_(1,37.405)_ = 0.0005, *p* = 0.982], no main effect of group [*F*_(1,39.291)_ = 0.5326, *p* = 0.469] and no main effect of time [*F*_(1,37.405)_ = 0.7808, *p* = 0.382].

#### ns-SCRs

Analysis revealed no significant group × time interaction [*F*_(1,34.555)_ = 0.0846, *p* = 0.773], and no main effect of group [*F*_(1,35.903)_ = 0.2140, *p* = 0.646]. A main effect of time was found [*F*_(1,34.416)_ = 6.5407, *p* = 0.015].

The results are illustrated in Supplementary Figure 2 and the means and standard deviations per group are reported in Table 3. Fixed effects estimates are reported in Supplementary Table 3.

**Table 3:** Means and standard deviations of the TMR and control groups on the ns-SCRs (number of events) and RMSSD (ms) levels during the generalization task periods (T0 and T4).

		**TMR group**	**Control group**
		***N* = 22**	***N* = 24**
T0	RMSSD	33.09 (13.47)	35.24 (14.13)
	ns-SCRs	12.28 (9.59)	13.57 (7.44)
T4	RMSSD	30.71 (17.11)	33.42 (11.78)
	ns-SCRs	8.61 (6.64)	8.75 (5.75)

*N = 46*.

### Association Between REM, TMR, and Stress Levels

#### RMSSD

**Figure 3:**
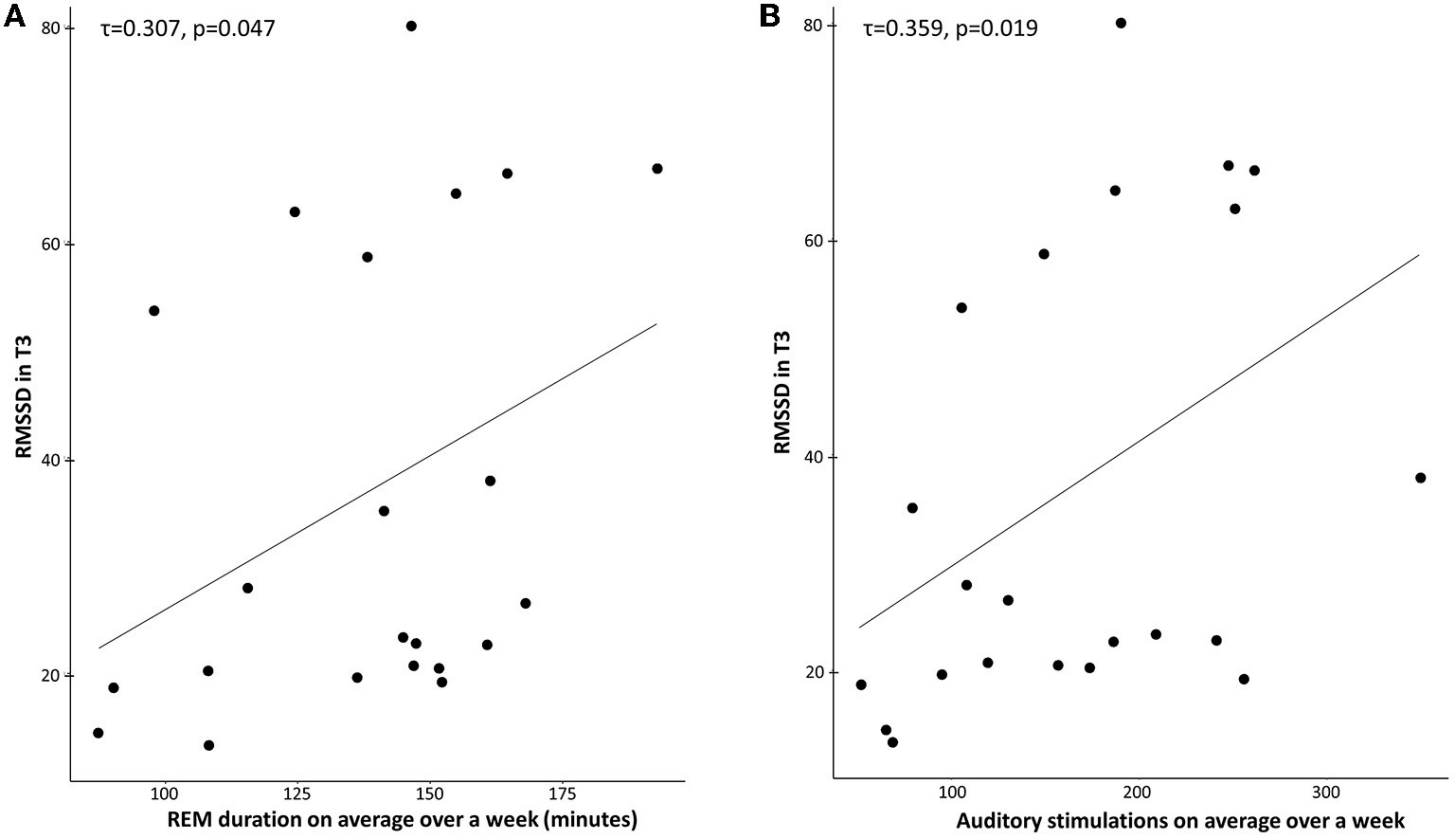
**(A)** Correlation between REM duration (minutes) and RMSSD in T3 for the TMR Group. Across participants, RMSSD scores were positively correlated with the mean REM duration over 1 week in the TMR group (τ = 0.307, *p* = 0.047). **(B)** Correlation between the number of stimulations on average over a week and RMSSD in T3 for the TMR group. Across participants, RMSSD scores were positively correlated with the mean number of stimulations in the TMR group (τ = 0.359, *p* = 0.019). Each dot indicates a participant.

RMSSD levels in T3 correlated with REM duration in the TMR group (*p* = 0.047, tau = 0.307; Figure 3A). The control group did not present this effect (*p* = 0.916, tau = −0.019). We used a Kendall correlation as the distribution of RMSSD was not normal (*p* = 0.001). After transformation of tau values to Pearson *r*-values (78), a Fisher *r*-to-*z* transformation test showed that the coefficients were significantly different (*p* = 0.046, *z* = 1.68). The correlation between REM sleep percentage and RMSSD in T3 was also significant for the TMR group (*p* = 0.024, tau = 0.347), but not for the control group (*p* = 0.834, tau = −0.035), with the two coefficients being significantly different (*p* = 0.026, *z* = 1.93).

A similar effect was observed for the correlation between the number of auditory stimulations and RMSSD in T3 (Figure 3B). The TMR group showed a significant effect (*p* = 0.019, tau = 0.359), but not the control group (*p* = 0.248, tau = −0.177). After transformation of tau values to Pearson *r*-values (78), a Fisher *r*-to-*z* transformation test showed that the coefficients were significantly different (*p* = 0.003, *z* = 2.77).

This significant positive correlation between REM sleep duration/ auditory stimulations and RMSSD was not present after one night of stimulations (T2) and no significant correlation was found between the stress variables in T2 or T3 and the other sleep stages (N1, N2, and N3).

When removing three potential outliers (all in the control group), the number of stimulations and RMSSD in T3 were still not correlated for this group (*p*-value = 0.2879, tau = −0.2371), neither did REM duration with RMSSD (*p*-value = 0.188, tau = −0.2987).

There was a positive correlation between the number of auditory stimulations (STIMs) and the duration of REM sleep over a week in the TMR group [*r* = 0.6458, *t*_(DF)_ = 22, *p* < 0.001]. We therefore tested whether the effect of STIMs on stress levels (RMSSD) at T3 was mediated by REM sleep. This was achieved with a bootstrap mediation test using 10000 approximate simulations [“mediate” function in R (79)]. The analysis revealed no significant direct effect of stimulations (*p* = 0.17) nor a significant mediated effect of stimulations through REM sleep (*p* = 0.54), but a significant total effect of stimulations (*p* = 0.015; Supplementary Table 4). These results indicate that REM sleep does not mediate the STIMs-RMSSD association.

#### SUDS

The correlation between REM duration and SUDS in T3 did not show any significant results for the TMR group (*p* = 0.295, *r* = 0.222) and the control group (*p* = 0.368, *r* = −0.191).

No significant results were found for the correlation between the number of auditory stimulations and SUDS in T3, in the TMR group (*p* = 0.522, *r* = 0.137) and the control group (*p* = 0.941, *r* = 0.015).

#### ns-SCR

The correlation between REM duration and ns-SCR in T3 did not show any significant results for the TMR group (*p* = 0.103, *r* = 0.396) and the control group (*p* = 0.546, *r* = −0.162).

Concerning the correlation of the number of auditory stimulations and ns-SCR in T3, no significant results were found in the TMR group (*p* = 0.39, *r* = 0.215) or the control group (*p* = 0.441, *r* = −0.207).

### Link Between Fear in Dreams and Stress in Wakefulness

No significant effect was found for the correlation between the change of fear in dreams (average of fear during the 2nd week with stimulations minus the 1st week without stimulations) and RMSSD in T3 for the TMR group (*p* = 0.1912, tau = −0.242) or the control group (*p* = 0.791, tau = −0.044).

**Figure 4:**
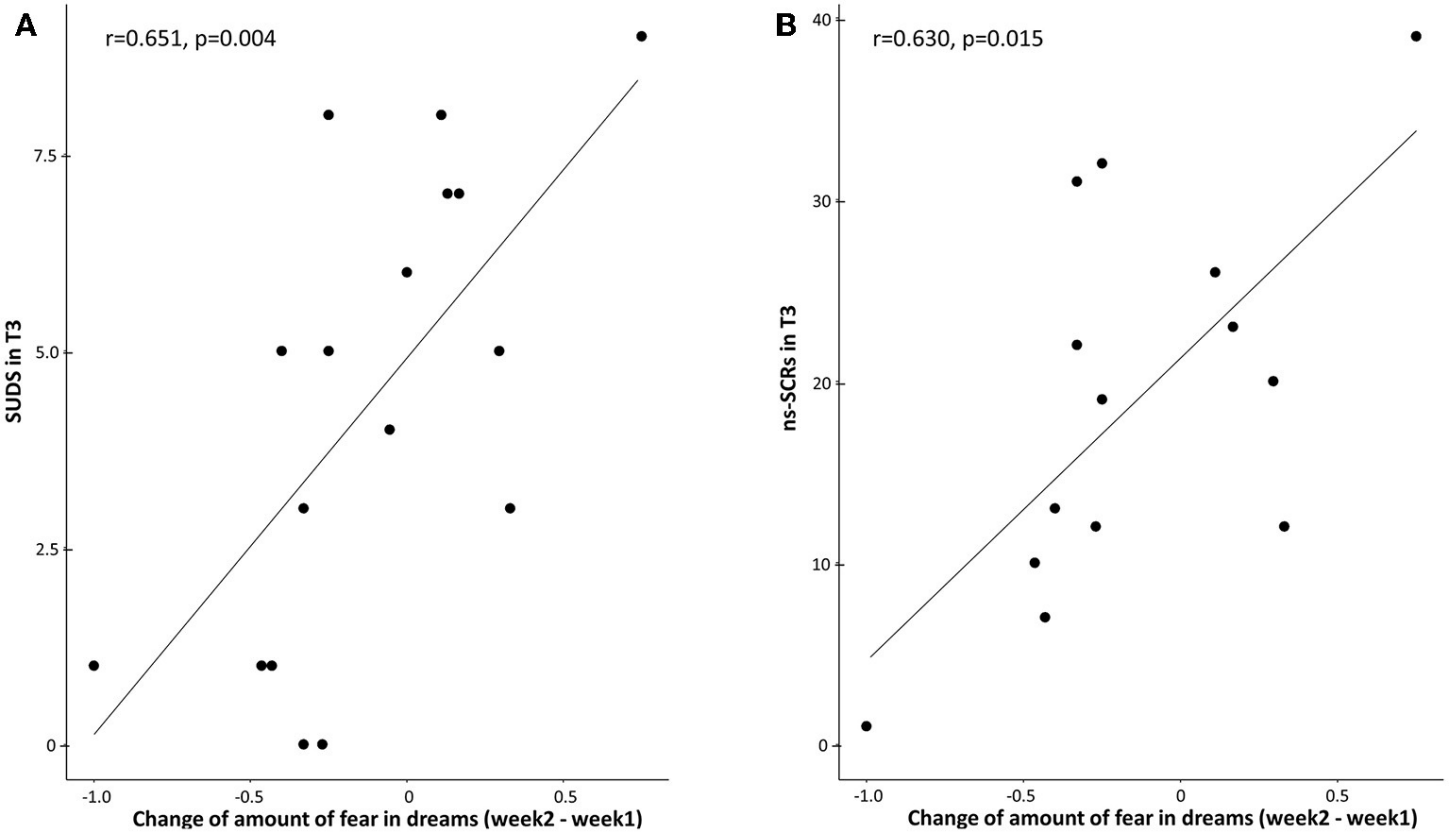
**(A)** Correlation between the change of fear in dreams (average of fear during the 2nd week with stimulations minus the 1st week without stimulations) and SUDS in T3 for the TMR group. Across participants, SUDS scores were positively correlated with the change of fear in the TMR group (*r* = 0.6513, *p* = 0.0046). **(B)** Correlation between the change of fear in dreams (average of fear during the 2nd week with stimulations minus the 1st week without stimulations) and ns-SCRs in T3 for the TMR group. Across participants, ns-SCRs scores were positively correlated with the change of fear in the TMR group (*r* = 0.6308, *p* = 0.0155). Each dot indicates a participant.

A significance is shown for the correlation between the change of fear and SUDS for the TMR group (*p* = 0.0046, *r* = 0.6513; Figure 4A), but not for the control group (*p* = 0.759, *r* = −0.0712). A Fisher *r*-to-*z* transformation test showed that the coefficients were significantly different (*p* = 0.009, *z* = −2.382).

A similar significant correlation between the change of fear in dreams and ns-SRCs was found for the TMR group (*p* = 0.0155, *r* = 0.6308; Figure 4B), but not for the control group (*p* = 0.7995, *r* = −0.0747). A Fisher *r*-to-*z* transformation test showed that the coefficients were significantly different (*p* = 0.011, *z* = −2.294).

## Discussion

The primary hypothesis of this study was to test if adding TMR during REM sleep in addition to standard exposure therapy would be more effective than exposure therapy alone in reducing anxiety in individuals suffering from SAD. TMR consisted in associating a sound with an isolated extinction period (i.e., the positive feedback of exposure therapy), and reactivating this memory by presenting the same sound during REM sleep. We did not observe lower anxiety levels in the TMR group, compared to the control group, during the preparation period preceding a speech after 8 days of auditory stimulation during REM, for any of our measures. However, we found that subjective anxiety was reduced across the experiment for both groups, indicating the efficiency of a small number of exposure therapy sessions in reducing social anxiety. In accordance with the depotentiation theory of REM sleep, secondary analyses also indicated that the number of auditory stimulations during REM sleep and REM sleep duration were positively correlated with RMSSD in the TMR group. A positive correlation between increased presence of fear in dreams and SUDS/ns-SCRs was also observed in the TMR group, reflecting a possible link between emotional content in dreams and waking stress levels in these patients. Such a result supports the idea that psychotherapeutic approaches targeting emotional dream content [e.g., reducing nightmare frequency and distress with Imagery Rehearsal Therapy (IRT)] (80), could be used to alleviate daytime depressive and anxiety symptoms too.

### Effect of Time

Among the three measures of anxiety, the SUDS was the only one to show an effect of time, indicating higher anxiety during the first preparation period (T1a) compared to the third (T2) and last (T3) ones in both groups. By contrast, the physiological measures did not show any modulation by time, probably because the small number of exposure therapy sessions in this protocol did not allow for such an improvement. The effect of time on subjective anxiety is in line with studies showing that exposure is efficient in reducing symptoms of anxiety disorders even without any other manipulation (10, 16, 81). However, strictly speaking, the lack of a control group without exposure therapy in the design of the current study cannot rule out that the significant reduction of subjective anxiety was not related to other factors (e.g., patient expectations; see limitations section below).

### Challenges to Isolate a Safety Memory

TMR during REM sleep in our experiment did not enhance the beneficial effect of exposure therapy on anxiety-related distress (SUDS). An explanation for these results could be that the sound in our experiment was not sufficiently associated with a safety memory (i.e., positive feedback). Indeed, when comparing anxiety levels of the preparation and feedback periods, physiological measures generally indicated similar levels between the two periods (Supplementary Material 3). Furthermore, anxiety levels were higher during feedbacks compared to the baseline periods, further demonstrating that the participants were still in a state of physiological stress during the feedback periods (Supplementary Material 3). Moreover, as patients with SAD show altered processing of feedback compared to healthy controls (82–84), positive feedback might not have been sufficient to change their expectations of a negative outcome.

Previous studies have associated a sound to aversive stimuli and presented this sound (conditioned stimulus, CS) during NREM sleep. This TMR procedure led to either a reduction in fear responses (85, 86) or increase of fear responses (87, 88). Protocol differences, such as reinforcement contingencies, relevance of the aversive stimuli or frequency of cueing during sleep, could explain such discrepancies (89). While these studies implicated memory reactivation during NREM sleep, and not REM sleep, these findings bring attention to the necessity to test multiple new experimental protocols in order to identify the mechanisms allowing sleep cueing to enhance extinction and those leading to opposite results. To this date, we do not know if consolidation of a positive memory during REM sleep would be more advantageous (90) than consolidation and subsequent depotentiation of a negative one (25).

### Effect of REM Sleep and TMR on Stress

We here found a positive correlation between REM duration over 1 week and RMSSD in T3 in the TMR group. Although pertaining to a secondary analysis and limited by its correlational nature, this result in a clinical population (i.e., SAD patients) supports the idea that REM sleep is beneficial for reducing some physiological manifestations of stress levels during wakefulness, in line with enhanced extinction (20, 22, 23) and emotional depotentiation (25, 40). Importantly, this correlation was not found for other sleep stages, in accordance with previous research (23–25, 39, 40). Indeed, results on the possible effects of N2 sleep and/or SWS on extinction learning are inconsistent. Some results show that N2 sleep is beneficial to reduce self-reported fear for spider phobia (17), while others show that N2/SWS (with or without TMR) does not enhance exposure therapy for spider phobia (91) or for SAD (19). As previously mentioned, TMR during SWS can also either enhance extinction (85, 86) or strengthen a fear memory (87, 88, 92). Thus, REM sleep would not strengthen the emotional tone of memories (93–96), but might instead consolidate negative memories while attenuating their emotional tone (24, 25, 39, 40, 97). REM sleep and dreaming (26, 98) might offer a permissive condition for the remodeling of negative experienced events.

The aforementioned links between REM sleep duration and RMSSD over eight nights are in line with previous findings that REM sleep measures averaged over several nights (trait-level effect of REM sleep) have a protective role in fear conditioning and suggest that REM deficiencies may predate the development of anxiety disorders and post-traumatic stress disorder (PTSD) (27, 99). Therefore, these results further substantiate previous evidence that REM sleep is a determinant factor in the modulation of anxiety and affective disorders (100), and that its structure and functional role should be protected in order to promote mental health (101).

Previous studies have explored the emotional role of REM sleep mainly in healthy participants. Our study suggests that REM sleep could benefit anxious patients but only after a TMR manipulation. Indeed, a positive effect of REM sleep on stress reduction was only seen in the TMR group of our study, where the sound has been associated with a previous waking emotional event. This observation may be related to the additional beneficial effects of (a) post-learning REM sleep on extinction, as REM sleep enhances such a function especially after a pre-sleep extinction training (23, 102), and (b) the further emotional depotentiation with TMR during REM sleep (39, 41). Notably, the aforementioned beneficial effects of TMR during REM sleep did not appear after one single night, but only after 1 week of stimulation at home. Therefore, apart from the trait-level effect of REM sleep mentioned above (27), repeated TMR stimulation over successive nights might be needed to permanently consolidate the formation of a new (i.e., initially labile) safety memory during sleep [e.g., make it hippocampus-independent (45)].

### Association Between Fear in Dreams and Anxiety in SAD Patients

The frequency of fear experienced in dreams correlated with the primary clinical outcome measure (SUDS) and the secondary psychophysiological outcome measure (ns-SCRs) in the TMR group (but not in the control group). The more these participants experienced fear in their dreams, the more they were stressed in T3, as measured with ns-SCRs and SUDS. In a previous study (26), we demonstrated that increased fear in dreams benefits reduced stress when healthy participants are exposed to fearful stimuli during wakefulness, and which was in accordance with an extinction function of dreaming in healthy participants (103). The results of the present study suggest that this fear extinction function of dreams (26, 103) might be deficient in clinical populations, such as anxious people here. For example, although healthy participants experiencing fearful dreams have higher mPFC activity in fearful situations in wakefulness (26), nightmare patients demonstrate a decreased mPFC activity during the viewing of negative pictures (104). We speculate that a similar failure of the fear extinction function of dreams can explain our result in patients with SAD, who also demonstrate a decreased mPFC activity and increased amygdala activity during social stressors (105). Whether there is a causal relationship between anxiety disorders and such a deficient extinction function of dreaming in these patients should be tested in future studies.

A strong link between dream emotions and daytime stress levels supports the idea that psychotherapeutic approaches aiming at modulating distressful emotions in dreams could also have an impact in daytime depressive and anxiety symptoms. For example, IRT is a cognitive-behavioral technique, where the nightmare sufferer changes the negative story line, toward a more positive ending, and rehearses the rewritten dream scenario during the day, which ultimately helps to reduce nightmares during sleep (106). This technique can be learned in one session (107) and practiced for 5–10 min per day while awake. It has been shown that IRT in PTSD patients can improve not only nightmare frequency and intensity, but daytime symptoms too (e.g., flashbacks, depressive symptoms, and dissociation) (80). The beneficial “halo” effect of IRT on daytime symptoms (and not only nightmares), may be related to its mechanism of action, which seems to implicate extinction learning (108). Our results support the idea that psychotherapeutic methods targeting negative emotions in dreams could be used to alleviate anxiety symptoms.

### Limitations

Apart from the challenges to isolate a safety memory (see Section Effect of Time), there are some other limitations in this study. First, all three measures of anxiety did not always concur (see Supplementary Table 5). On the one hand, a dissociation between physiological and subjective measures of anxiety is very common (19, 109) and seems to reflect a different response of the autonomic nervous system and of subjective (conscious) experience to perceived stress (19, 110). A dissociation between heart rate and skin conductance reactions is also found in SAD (72). On the other hand, this dissociation may be related to the use of a VR setting for the measurement of stress during exposure therapy. While such a setting allows for realistic and controlled exposure to feared stimuli, it may have reduced the participants' immersion in the feared situation (63, 111). Comparing subjective assessments with different physiological measurements is needed to have more insight on the implication of different systems in stress responses (112). Another limitation, linked to the assessment of anxiety, relates to the physiological measures (RMSSD, ns-SCRs). While in our study we use RMSSD and ns-SCRs to assess anxiety levels, the fact that these measures can be modulated by other factors should not be overlooked. Indeed, RMSSD and ns-SCRs reflect some physiological manifestations of stress, but have also been linked to depression, worry, and measures of inflammation or cognitive functions involving the prefrontal cortex (113, 114).

Moreover, it is possible that a reduction of sleep quantity may have accounted for the negative results of our study. Indeed, total sleep time (TST) and N2 sleep were decreased during the week at home compared to the habituation night (see Supplementary Material 4, Supplementary Table 1). Moreover, TMR participants were almost significantly (*p* = 0.08) less vigilant compared to those of the control group, as measured by the PVT before the last VR session at T3 (see Supplementary Table 1), while HRV is sensitive to sleep deprivation (115). This means that, even though REM sleep and auditory stimulations had a positive effect on stress within this group, sleep conditions may be a more important determinant for the participant's wellbeing and stress levels (although the relationship between stress and sleep is bidirectional) (116). Indeed, TMR seems to lose its positive effect when sleep is compromised (117), while sufficient sleep predicts treatment outcome of exposure therapy in patients with SAD (118, 119). Our results hence suggest that the strengthening of a safety memory during REM sleep with TMR can be helpful to reduce stress only when controlling for sleep disruption, in line with previous research (117). Indeed, in a recent paper (101), the authors stated that the beneficial effects of REM sleep on extinction are not noticeable if sleep is disturbed.

Finally, this experiment lacks a SAD group without any stimulation or a SAD group without exposure therapy or a SAD group with exposure therapy but no positive feedback. Therefore, although we know that exposure therapy promotes extinction (5) and that REM sleep enhances extinction in healthy participants after an extinction task compared to wakefulness or NREM sleep (22, 23), we cannot safely conclude from our study that TMR during REM sleep or a positive feedback period of exposure therapy would add an extra benefit in extinction processes. Indeed, previous studies indicate the presence of a ceiling effect of the highly effective exposure therapy or of REM sleep alone in extinction processes (22, 91). Moreover, to control for any unspecific effect of the sound played during the positive feedback phase of the exposure therapy (e.g., S1, in the TMR group), future studies may use a different sound (e.g., S2) played during the feedback phase in the control group, while the same sound (S1) would be played during sleep of both groups. This sound (S2) in the control group would need to be similar in terms of valence and intensity to the sound (S1) in the TMR group, but sufficiently different to minimize generalization between the two sounds in the control group. With such a design, the two groups would be similar in the total auditory stimuli they receive during both wakefulness and sleep and potential confounds of playing a sound only in the TMR group in wakefulness would be avoided.

As a technical note, in the present study, we applied TMR during REM sleep, irrespective of REM tonic or phasic states. Yet, for the purpose of extinction consolidation, TMR should ideally be performed during phasic REM sleep, as phasic P-waves seem important for the retention of fear extinction memory occurring after the acquisition of fear extinction learning (102). Therefore, future technical development of TMR applied during REM may aim at achieving selective stimulations during phasic REM.

### Future Perspectives

To the best of our knowledge, this is the first TMR study exploring the links between REM sleep, extinction and anxiety in a clinical population. Although this study does not allow to ascertain a positive effect of TMR on SAD, TMR remains an efficient tool for the reprocessing of emotional memories (37, 39), and further research with TMR could be the key to develop efficient therapies for SAD or other emotional disorders implicating deficient extinction learning (90). TMR application during sleep may avoid certain disadvantages of traditional exposure therapies during wakefulness, such as worsening mood and anxiety during the recall of painful experiences. By deploying and popularizing easy-to-use devices at home in order to enhance the consolidation of safety memories, these therapies could reach a big part of the general population and lead to new approaches for promoting emotional wellbeing.

Future studies should better characterize the nature of the link between anxiety disorders and the deficient extinction function of dreaming, and they could also test whether psychotherapeutic methods targeting distressful dreams, such as IRT, can be helpful for treating anxiety disorders as well.

## Data Availability Statement

The original contributions presented in the study are included in the article/Supplementary Material, further inquiries can be directed to the corresponding author.

## Ethics Statement

The studies involving human participants were reviewed and approved by Ethical Committee of the Canton of Geneva, Switzerland (Commission Cantonale d'Ethique de la Recherche sur l'être humain). The patients/participants provided their written informed consent to participate in this study.

## Author Contributions

SS and LP designed the experiments. FB, PH, FG, CP, and LP conducted the experiments and analyzed the data. FB, PH, FG, CP, SD, SS, and LP wrote the paper. All authors contributed to the article and approved the submitted version.

## Funding

This study was supported by the Medical Direction of University Hospitals of Geneva (PRD 18-2019-I to LP) and the Swiss National Science Foundation (grants 320030_159862 and 320030_182589 to SS, grant CRSK-3_190722 to LP). Registration number NCT05261659. Open access funding was provided by the University of Geneva.

## Conflict of Interest

The authors declare that the research was conducted in the absence of any commercial or financial relationships that could be construed as a potential conflict of interest.

## Publisher's Note

All claims expressed in this article are solely those of the authors and do not necessarily represent those of their affiliated organizations, or those of the publisher, the editors and the reviewers. Any product that may be evaluated in this article, or claim that may be made by its manufacturer, is not guaranteed or endorsed by the publisher.

## References

[1] Leichsenring F Leweke F. Social Anxiety Disorder. New Engl J Med. (2017) 376:2255–64. 10.1056/NEJMcp1614701 28591542

[2] Stein MB Stein DJ. Social Anxiety Disorder. Lancet. (2008) 371:1115–25. 10.1016/S0140-6736(08)60488-2 18374843

[3] Bandelow B Michaelis S. Epidemiology of anxiety disorders in the 21st century. Dialogues Clin Neurosci. (2015) 17:327–35. 10.31887/DCNS.2015.17.3/bbandelow 26487813 PMC4610617

[4] Morrison AS Heimberg RG. Social anxiety and social anxiety disorder. Annu Rev Clin Psycho. (2013) 9:249–74. 10.1146/annurev-clinpsy-050212-185631 23537485

[5] Craske MG Hermans D Vervliet B. State-of-the-art and future directions for extinction as a translational model for fear and anxiety. Philos Trans R Soc Lond B Biol Sci. (2018) 25:373. 10.1098/rstb.2017.0025 29352025 PMC5790824

[6] Graham BM Milad MR. The study of fear extinction: implications for anxiety disorders. Am J Psychiatry. (2011) 168:1255–65. 10.1176/appi.ajp.2011.11040557 21865528 PMC4118766

[7] Neumann DL Waters AM. The use of an unpleasant sound as an unconditional stimulus in a human aversive Pavlovian conditioning procedure. Biol Psychol. (2006) 73:175–85. 10.1016/j.biopsycho.2006.03.004 16698165

[8] Bouton ME. Context, ambiguity, and unlearning: sources of relapse after behavioral extinction. Biol Psychiatry. (2002) 52:976–86. 10.1016/S0006-3223(02)01546-9 12437938

[9] Craske MG Treanor M Conway CC Zbozinek T Vervliet B. Maximizing exposure therapy: an inhibitory learning approach. Behav Res Ther. (2014) 58:10–23. 10.1016/j.brat.2014.04.006 24864005 PMC4114726

[10] Kaczkurkin AN Foa EB. Cognitive-behavioral therapy for anxiety disorders: an update on the empirical evidence. Dialogues Clin Neuro. (2015) 17:337–46. 10.31887/DCNS.2015.17.3/akaczkurkin 26487814 PMC4610618

[11] Bouchard S Dumoulin S Robillard G Guitard T Klinger E Forget H. Virtual reality compared with *in vivo* exposure in the treatment of social anxiety disorder: a three-arm randomised controlled trial. Br J Psychiatry. (2017) 210:276–83. 10.1192/bjp.bp.116.184234 27979818

[12] Carl E Stein AT Levihn-Coon A Pogue JR Rothbaum B Emmelkamp P. Virtual reality exposure therapy for anxiety and related disorders: a meta-analysis of randomized controlled trials. J Anxiety Disord. (2019) 61:27–36. 10.1016/j.janxdis.2018.08.003 30287083

[13] Soravia LM Heinrichs M Winzeler L Fisler M Schmitt W Horn H. Glucocorticoids enhance *in vivo* exposure-based therapy of spider phobia. Depress Anxiety. (2014) 31:429–35. 10.1002/da.22219 24265104

[14] Raij T Nummenmaa A Marin MF Porter D Furtak S Setsompop K. Prefrontal cortex stimulation enhances fear extinction memory in humans. Biol Psychiatry. (2018) 84:129–37. 10.1016/j.biopsych.2017.10.022 29246436 PMC5936658

[15] Hartanto D Kampmann IL Morina N Emmelkamp PG Neerincx MA Brinkman WP. Controlling social stress in virtual reality environments. PLoS ONE. (2014) 9:e92804. 10.1371/journal.pone.0092804 24671006 PMC3966821

[16] Borgeat F Stankovic M Khazaal Y Rouget BW Baumann MC Riquier F. Does the form or the amount of exposure make a difference in the cognitive-behavioral therapy treatment of social phobia? J Nerv Ment Dis. (2009) 197:507–13. 10.1097/NMD.0b013e3181aacc08 19597358

[17] Kleim B Wilhelm FH Temp L Margraf J Wiederhold BK Rasch B. Sleep enhances exposure therapy. Psychol Med. (2014) 44:1511–9. 10.1017/S0033291713001748 23842278

[18] Pace-Schott EF Verga PW Bennett TS Spencer RM. Sleep promotes consolidation and generalization of extinction learning in simulated exposure therapy for spider fear. J Psychiatr Res. (2012) 46:1036–44. 10.1016/j.jpsychires.2012.04.015 22578824 PMC3392441

[19] Pace-Schott EF Bottary RM Kim SY Rosencrans PL Vijayakumar S Orr SP. Effects of post-exposure naps on exposure therapy for social anxiety. Psychiatry Res. (2018) 270:523–30. 10.1016/j.psychres.2018.10.015 30340182 PMC6292728

[20] Spoormaker VI Schroter MS Andrade KC Dresler M Kiem SA Goya-Maldonado R. Effects of rapid eye movement sleep deprivation on fear extinction recall and prediction error signaling. Hum Brain Mapp. (2012) 33:2362–76. 10.1002/hbm.21369 21826762 PMC6870311

[21] Spoormaker VI Sturm A Andrade KC Schroter MS Goya-Maldonado R Holsboer F. The neural correlates and temporal sequence of the relationship between shock exposure, disturbed sleep and impaired consolidation of fear extinction. J Psychiatr Res. (2010) 44:1121–8. 10.1016/j.jpsychires.2010.04.017 20471033

[22] Pace-Schott EF Tracy LE Rubin Z Mollica AG Ellenbogen JM Bianchi MT. Interactions of time of day and sleep with between-session habituation and extinction memory in young adult males. Exp Brain Res. (2014) 232:1443–58. 10.1007/s00221-014-3829-9 24481663 PMC4013206

[23] Menz MM Rihm JS Buchel C. Rem sleep is causal to successful consolidation of dangerous and safety stimuli and reduces return of fear after extinction. J Neurosci. (2016) 36:2148–60. 10.1523/JNEUROSCI.3083-15.2016 26888926 PMC6602040

[24] Gujar N McDonald SA Nishida M Walker MP. A role for rem sleep in recalibrating the sensitivity of the human brain to specific emotions. Cereb Cortex. (2011) 21:115–23. 10.1093/cercor/bhq064 20421251 PMC3000566

[25] van der Helm E Yao J Dutt S Rao V Saletin JM Walker MP. Rem sleep depotentiates amygdala activity to previous emotional experiences. Curr Biol. (2011) 21:2029–32. 10.1016/j.cub.2011.10.052 22119526 PMC3237718

[26] Sterpenich V Perogamvros L Tononi G Schwartz S. Fear in dreams and in wakefulness: evidence for day/night affective homeostasis. Hum Brain Mapp. (2020) 41:840–50. 10.1002/hbm.24843 31663236 PMC7267911

[27] Lerner I Lupkin SM Sinha N Tsai A Gluck MA. Baseline levels of rapid eye movement sleep may protect against excessive activity in fear-related neural circuitry. J Neurosci. (2017) 37:11233–44. 10.1523/JNEUROSCI.0578-17.2017 29061703 PMC6596812

[28] Lerner I Lupkin SM Corter JE Peters SE Cannella LA Gluck MA. The influence of sleep on emotional and cognitive processing is primarily trait- (but not state-) dependent. Neurobiol Learn Mem. (2016) 134:275–86. 10.1016/j.nlm.2016.07.032 27481220

[29] Oudiette D Paller KA. Upgrading the sleeping brain with targeted memory reactivation. Trends Cogn Sci. (2013) 17:142–9. 10.1016/j.tics.2013.01.006 23433937

[30] Rasch B Buchel C Gais S Born J. Odor cues during slow-wave sleep prompt declarative memory consolidation. Science. (2007) 315:1426–9. 10.1126/science.1138581 17347444

[31] Diekelmann S. Sleep for cognitive enhancement. Front Syst Neurosci. (2014) 8:46. 10.3389/fnsys.2014.00046 24765066 PMC3980112

[32] Rudoy JD Voss JL Westerberg CE Paller KA. Strengthening individual memories by reactivating them during sleep. Science. (2009) 326:1079. 10.1126/science.1179013 19965421 PMC2990343

[33] Klinzing JG Diekelmann S. Cued memory reactivation: a tool to manipulate memory consolidation during sleep. In: Drinhenberg HC, editor, Handbook of Behavioral Neuroscience. London: Elsevier (2019). p. 471–88. 10.1016/B978-0-12-813743-7.00031-1

[34] Schouten DI Pereira SI Tops M Louzada FM. State of the art on targeted memory reactivation: sleep your way to enhanced cognition. Sleep Med Rev. (2017) 32:123–31. 10.1016/j.smrv.2016.04.002 27296303

[35] Guerrien A Dujardin K Mandai O Sockeel P Leconte P. Enhancement of memory by auditory stimulation during postlearning rem sleep in humans. Physiol Behav. (1989) 45:947–50. 10.1016/0031-9384(89)90219-9 2780879

[36] Smith C Weeden K. Post training rems coincident auditory stimulation enhances memory in humans. Psychiatr J Univ Ott. (1990) 15:85–90. 2374793

[37] Sterpenich V Schmidt C Albouy G Matarazzo L Vanhaudenhuyse A Boveroux P. Memory reactivation during rapid eye movement sleep promotes its generalization and integration in cortical stores. Sleep. (2014) 37:1061–75. 10.5665/sleep.3762 24882901 PMC4015380

[38] Ashton JE Cairney SA Gaskell MG. No effect of targeted memory reactivation during slow-wave sleep on emotional recognition memory. J Sleep Res. (2018) 27:129–37. 10.1111/jsr.12542 28493346

[39] Rihm JS Rasch B. Replay of conditioned stimuli during late rem and stage N2 sleep influences affective tone rather than emotional memory strength. Neurobiol Learn Mem. (2015) 122:142–51. 10.1016/j.nlm.2015.04.008 25933506

[40] Walker MP van der Helm E. Overnight therapy? The role of sleep in emotional brain processing. Psychol Bull. (2009) 135:731–48. 10.1037/a0016570 19702380 PMC2890316

[41] Hutchison IC Pezzoli S Tsimpanouli ME Abdellahi MEA Pobric G Hulleman J. Targeted memory reactivation in rem but not Sws selectively reduces arousal responses. Commun Biol. (2021) 4:404. 10.1038/s42003-021-01854-3 33767319 PMC7994443

[42] Diekelmann S Born J. The memory function of sleep. Nat Rev Neurosci. (2010) 11:114–26. 10.1038/nrn2762 20046194

[43] Rasch B Born J. About sleep's role in memory. Physiol Rev. (2013) 93:681–766. 10.1152/physrev.00032.2012 23589831 PMC3768102

[44] Maren S Chang CH. Recent fear is resistant to extinction. Proc Natl Acad Sci USA. (2006) 103:18020–5. 10.1073/pnas.0608398103 17090669 PMC1693865

[45] Frankland PW Bontempi B. The organization of recent and remote memories. Nat Rev Neurosci. (2005) 6:119–30. 10.1038/nrn1607 15685217

[46] Carrillo E Moya-Albiol L Gonzalez-Bono E Salvador A Ricarte J Gomez-Amor J. Gender differences in cardiovascular and electrodermal responses to public speaking task: the role of anxiety and mood states. Int J Psychophysiol. (2001) 42:253–64. 10.1016/S0167-8760(01)00147-7 11812392

[47] Gonzalez-Bono E Moya-Albiol L Salvador A Carrillo E Ricarte J Gomez-Amor J. Anticipatory autonomic response to a public speaking task in women: the role of trait anxiety. Biol Psychol. (2002) 60:37–49. 10.1016/S0301-0511(02)00008-X 12100844

[48] Tardy CH Allen MT. Moderators of cardiovascular reactivity to speech: discourse production and group variations in blood pressure and pulse rate. Int J Psychophysiol. (1998) 29:247–54. 10.1016/S0167-8760(98)00003-8 9666379

[49] American Psychiatric Association. Diagnostic and Statistical Manual of Mental Disorders. 5th ed. Arlington, VA: American Psychiatric Publishing (2013). 10.1176/appi.books.9780890425596

[50] Arnal PJ Thorey V Debellemaniere E Ballard ME Bou Hernandez A Guillot A. The dreem headband compared to polysomnography for electroencephalographic signal acquisition and sleep staging. Sleep. (2020) 43:zsaa097. 10.1093/sleep/zsaa097 32433768 PMC7751170

[51] Liebowitz MR. Social phobia. Mod Probl Pharmacopsychiatry. (1987) 22:141–73. 10.1159/000414022 2885745

[52] Buysse DJ Reynolds CF 3rd Monk TH Berman SR Kupfer DJ. The Pittsburgh sleep quality index: a new instrument for psychiatric practice and research. Psychiatry Res. (1989) 28:193–213. 10.1016/0165-1781(89)90047-4 2748771

[53] Beck AT Epstein N Brown G Steer RA. An inventory for measuring clinical anxiety: psychometric properties. J Consult Clin Psychol. (1988) 56:893–7. 10.1037/0022-006X.56.6.893 3204199

[54] Beck AT Steer RA Brown GK. Manual for the Beck Depression Inventory-Ii. San Antonio, TX: Psychological Corporation (1996). 10.1037/t00742-000

[55] Morin CM Belleville G Belanger L Ivers H. The insomnia severity index: psychometric indicators to detect insomnia cases and evaluate treatment response. Sleep. (2011) 34:601–8. 10.1093/sleep/34.5.601 21532953 PMC3079939

[56] Sheehan DV. Mini Neuropsychiatric International Interview 7.0 (Mini 7.0). Jacksonville, FL: Medical Outcomes Systems, Jacksonville (2015).

[57] Basner M Dinges DF. Maximizing sensitivity of the psychomotor vigilance test (Pvt) to sleep loss. Sleep. (2011) 34:581–91. 10.1093/sleep/34.5.581 21532951 PMC3079937

[58] Ellis BW Johns MW Lancaster R Raptopoulos P Angelopoulos N Priest RG. The St. Mary's hospital sleep questionnaire: a study of reliability. Sleep. (1981) 4:93–7. 10.1093/sleep/4.1.93 7232974

[59] Alvares GA Quintana DS Kemp AH Van Zwieten A Balleine BW Hickie IB. Reduced heart rate variability in social anxiety disorder: associations with gender and symptom severity. PLoS ONE. (2013) 8:e70468. 10.1371/journal.pone.0070468 23936207 PMC3728204

[60] Pulopulos MM Vanderhasselt MA De Raedt R. Association between changes in heart rate variability during the anticipation of a stressful situation and the stress-induced cortisol response. Psychoneuroendocrinology. (2018) 94:63–71. 10.1016/j.psyneuen.2018.05.004 29758470 PMC5967249

[61] Wendt J Neubert J Koenig J Thayer JF Hamm AO. Resting heart rate variability is associated with inhibition of conditioned fear. Psychophysiology. (2015) 52:1161–6. 10.1111/psyp.12456 26095980

[62] Boucsein W Fowles DC Grimnes S Ben-Shakhar G Roth WT Dawson ME. Publication recommendations for electrodermal measurements. Psychophysiology. (2012) 49:1017–34. 10.1111/j.1469-8986.2012.01384.x 22680988

[63] Wilhelm FH Pfaltz MC Gross JJ Mauss IB Kim SI Wiederhold BK. Mechanisms of virtual reality exposure therapy: the role of the behavioral activation and behavioral inhibition systems. Appl Psychophysiol Biofeedback. (2005) 30:271–84. 10.1007/s10484-005-6383-1 16167191

[64] Wolpe J. The Practice of Behavior Therapy. 4th ed New York: Pergamon Press (1990).

[65] Shaffer F Ginsberg JP. An overview of heart rate variability metrics and norms. Front Public Health. (2017) 5:258. 10.3389/fpubh.2017.00258 29034226 PMC5624990

[66] Chalmers JA Quintana DS Abbott MJ Kemp AH. Anxiety disorders are associated with reduced heart rate variability: a meta-analysis. Front Psychiatry. (2014) 5:80. 10.3389/fpsyt.2014.00080 25071612 PMC4092363

[67] Laborde S Mosley E Thayer JF. Heart rate variability and cardiac vagal tone in psychophysiological research - recommendations for experiment planning, data analysis, and data reporting. Front Psychol. (2017) 8:213. 10.3389/fpsyg.2017.00213 28265249 PMC5316555

[68] Madison A Vasey M Emery CF Kiecolt-Glaser JK. Social anxiety symptoms, heart rate variability, and vocal emotion recognition in women: evidence for parasympathetically-mediated positivity bias. Anxiety Stress Coping. (2021) 34:243–57. 10.1080/10615806.2020.1839733 33156720

[69] Petrescu L Petrescu C Mitrut O Moise G Moldoveanu A Moldoveanu F. Integrating biosignals measurement in virtual reality environments for anxiety detection. Sensors. (2020) 20:47088. 10.3390/s20247088 33322014 PMC7763206

[70] Wolpe J. The Practice of Behavior Therapy. New York, NY: Pergamon Press (1969).

[71] Cornwell BR Johnson L Berardi L Grillon C. Anticipation of public speaking in virtual reality reveals a relationship between trait social anxiety and startle reactivity. Biol Psychiat. (2006) 59:664–6. 10.1016/j.biopsych.2005.09.015 16325155

[72] Iffland B Sansen LM Catani C Neuner F. Rapid heartbeat, but dry palms: reactions of heart rate and skin conductance levels to social rejection. Front Psychol. (2014) 5:956. 10.3389/fpsyg.2014.00956 25221535 PMC4148623

[73] Berry RB Brooks R Gamaldo CE Hardling S Marcus C Vaughn B. The Aasm Manual for the Scoring of Sleep and Associated Events. Rules, Terminology and Technical Specifications. Darien, IL: American Academy of Sleep Medicine (2012).

[74] Gaebler M Daniels JK Lamke JP Fydrich T Walter H. Heart rate variability and its neural correlates during emotional face processing in social anxiety disorder. Biol Psychol. (2013) 94:319–30. 10.1016/j.biopsycho.2013.06.009 23831279

[75] Fitzmaurice GM Laird NM Ware JH. Applied Longitudinal Analysis: Hoboken, NJ: Wiley-Interscience (2004).

[76] Bates D Mächler M Bolker B Walker SC. Fitting linear mixed-effects models using Lme4. J Stat Softw. (2015) 67:1–48. 10.18637/jss.v067.i01

[77] Kuznetsova A Brockhoff PB Christensen RHB. Lmertest package: tests in linear mixed effects models. J Stat Softw. (2017) 82:1–26. 10.18637/jss.v082.i13

[78] Walker DA. Converting Kendall's tau for correlational or meta-analytic analyses. J Mod Appl Stat Methods. (2003) 2:525–30. 10.22237/jmasm/1067646360

[79] Tingley D Yamamoto T Hirose K Keele L Imai K. Mediation: R package for causal mediation analysis. J Stat Softw. (2014) 59:1–38. 10.18637/jss.v059.i0526917999

[80] Krakow B Hollifield M Johnston L Koss M Schrader R Warner TD. Imagery rehearsal therapy for chronic nightmares in sexual assault survivors with posttraumatic stress disorder - a randomized controlled trial. J Am Med Assoc. (2001) 286:537–45. 10.1001/jama.286.5.537 11476655

[81] Klinger E Bouchard S Legeron P Roy S Lauer F Chemin I. Virtual reality therapy versus cognitive behavior therapy for social phobia: a preliminary controlled study. Cyberpsychol Behav. (2005) 8:76–88. 10.1089/cpb.2005.8.76 15738695

[82] Glazier BL Alden LE. Social anxiety disorder and memory for positive feedback. J Abnorm Psychol. (2019) 128:228–33. 10.1037/abn0000407 30702303

[83] Koban L Schneider R Ashar YK Andrews-Hanna JR Landy L Moscovitch DA. Social anxiety is characterized by biased learning about performance and the self. Emotion. (2017) 17:1144–55. 10.1037/emo0000296 28358557 PMC5623172

[84] Voegler R Peterburs J Bellebaum C Straube T. Modulation of feedback processing by social context in social anxiety disorder (sad)-an event-related potentials (Erps) study. Sci Rep. (2019) 9:4795. 10.1038/s41598-019-41268-0 30886233 PMC6423138

[85] Hauner KK Howard JD Zelano C Gottfried JA. Stimulus-specific enhancement of fear extinction during slow-wave sleep. Nat Neurosci. (2013) 16:1553–5. 10.1038/nn.3527 24056700 PMC3818116

[86] He J Sun HQ Li SX Zhang WH Shi J Ai SZ. Effect of conditioned stimulus exposure during slow wave sleep on fear memory extinction in humans. Sleep. (2015) 38:423–31. 10.5665/sleep.4502 25348121 PMC4335533

[87] Barnes DC Wilson DA. Slow-wave sleep-imposed replay modulates both strength and precision of memory. J Neurosci. (2014) 34:5134–42. 10.1523/JNEUROSCI.5274-13.2014 24719093 PMC3983797

[88] Rolls A Makam M Kroeger D Colas D de Lecea L Heller HC. Sleep to forget: interference of fear memories during sleep. Mol Psychiatry. (2013) 18:1166–70. 10.1038/mp.2013.121 24081009 PMC5036945

[89] Diekelmann S Born J. Cueing fear memory during sleep–to extinguish or to enhance fear? Sleep. (2015) 38:337–9. 10.5665/sleep.4484 25669194 PMC4335524

[90] Schwartz S Clerget A Perogamvros L. Combined treatment of nightmares with targeted memory reactivation and imagery rehearsal therapy: a randomized controlled trial. medRxiv. (2022). 10.1101/2022.02.17.22270256 36306786

[91] Rihm JS Sollberger SB Soravia LM Rasch B. Re-presentation of olfactory exposure therapy success cues during non-rapid eye movement sleep did not increase therapy outcome but increased sleep spindles. Front Hum Neurosci. (2016) 10:340. 10.3389/fnhum.2016.00340 27445775 PMC4928007

[92] Ai SZ Dai XJ. Causal role of rapid-eye-movement sleep on successful memory consolidation of fear extinction. J Thorac Dis. (2018) 10:1214–6. 10.21037/jtd.2018.01.163 29707269 PMC5906322

[93] Baran B Pace-Schott EF Ericson C Spencer RMC. Processing of emotional reactivity and emotional memory over sleep. J Neurosci. (2012) 32:1035–42. 10.1523/JNEUROSCI.2532-11.2012 22262901 PMC3548452

[94] Jones BJ Spencer RMG. Sleep preserves subjective and sympathetic emotional response of memories. Neurobiol Learn Mem. (2019) 166:107096. 10.1016/j.nlm.2019.107096 31585163 PMC7927201

[95] Wagner U Fischer S Born J. Changes in emotional responses to aversive pictures across periods rich in slow-wave sleep versus rapid eye movement sleep. Psychosom Med. (2002) 64:627–34. 10.1097/00006842-200207000-00013 12140353

[96] Werner GG Schabus M Blechert J Kolodyazhniy V Wilhelm FH. Pre-to postsleep change in psychophysiological reactivity to emotional films: late-night rem sleep is associated with attenuated emotional processing. Psychophysiology. (2015) 52:813–25. 10.1111/psyp.12404 25588962

[97] Cunningham TJ Crowell CR Alger SE Kensinger EA Villano MA Mattingly SM. Psychophysiological arousal at encoding leads to reduced reactivity but enhanced emotional memory following sleep. Neurobiol Learn Mem. (2014) 114:155–64. 10.1016/j.nlm.2014.06.002 24952130

[98] Vallat R Chatard B Blagrove M Ruby P. Characteristics of the memory sources of dreams: a new version of the content-matching paradigm to take mundane and remote memories into account. PLoS ONE. (2017) 12:185262. 10.1371/journal.pone.0185262 29020066 PMC5636081

[99] Pace-Schott EF Germain A Milad MR. Sleep and rem sleep disturbance in the pathophysiology of Ptsd: the role of extinction memory. Biol Mood Anxiety Disord. (2015) 5:3. 10.1186/s13587-015-0018-9 26034578 PMC4450835

[100] Wassing R Lakbila-Kamal O Ramautar JR Stoffers D Schalkwijk F Van Someren EJW. Restless rem sleep impedes overnight amygdala adaptation. Curr Biol. (2019) 29:2351–8 e4. 10.1016/j.cub.2019.06.034 31303489

[101] Davidson P Pace-Schott E. The role of sleep in fear learning and memory. Curr Opin Psychol. (2020) 34:32–6. 10.1016/j.copsyc.2019.08.016 31568938 PMC7048665

[102] Datta S O'Malley MW. Fear extinction memory consolidation requires potentiation of pontine-wave activity during rem sleep. J Neurosci. (2013) 33:4561–9. 10.1523/JNEUROSCI.5525-12.2013 23467372 PMC3595135

[103] Nielsen T Levin R. Nightmares: a new neurocognitive model. Sleep Med Rev. (2007) 11:295–310. 10.1016/j.smrv.2007.03.004 17498981

[104] Marquis L Blanchette-Carriere C Carr M Julien S Paquette T Nielsen T. Decreased activity in medial prefrontal cortex and anterior cingulate cortex in idiopathic nightmare sufferers during wakefulness. Sleep. (2016) 39:A226–7.

[105] Marazziti D Abelli M Baroni S Carpita B Ramacciotti CE Dell'Osso L. Neurobiological correlates of social anxiety disorder: an update. Cns Spectrums. (2015) 20:100–11. 10.1017/S109285291400008X 24571962

[106] Krakow B Zadra A. Imagery rehearsal therapy: principles and practice. Sleep Med Clin. (2010) 5:289–98. 10.1016/j.jsmc.2010.01.00426522908

[107] Germain A Nielsen T. Impact of imagery rehearsal treatment on distressing dreams, psychological distress, and sleep parameters in nightmare patients. Behav Sleep Med. (2003) 1:140–54. 10.1207/S15402010BSM0103_2 15600218

[108] Rousseau A Belleville G. The mechanisms of action underlying the efficacy of psychological nightmare treatments: a systematic review and thematic analysis of discussed hypotheses. Sleep Med Rev. (2018) 39:122–33. 10.1016/j.smrv.2017.08.004 29056416

[109] Campbell J Ehlert U. Acute psychosocial stress: does the emotional stress response correspond with physiological responses? Psychoneuroendocrinology. (2012) 37:1111–34. 10.1016/j.psyneuen.2011.12.010 22260938

[110] Grillon C. D-cycloserine facilitation of fear extinction and exposure-based therapy might rely on lower-level, automatic mechanisms. Biol Psychiatry. (2009) 66:636–41. 10.1016/j.biopsych.2009.04.017 19520359 PMC2752328

[111] Kritikos J Tzannetos G Zoitaki C Poulopoulou S Koutsouris PD. Anxiety detection from electrodermal activity sensor with movement interaction during virtual reality simulation. In: International IEEE/EMBS Conference on Neural Engineering, NER; March 2019. San Francisco, CA. (2019). p. 1–6.

[112] Kerous B Bartecek R Roman R Sojka P Becev O Liarokapis F. Examination of electrodermal and cardio-vascular reactivity in virtual reality through a combined stress induction protocol. J Amb Intel Hum Comp. (2020) 11:6033–42. 10.1007/s12652-020-01858-7

[113] McCraty R Shaffer F. Heart rate variability: new perspectives on physiological mechanisms, assessment of self-regulatory capacity, and health risk. Glob Adv Health Med. (2015) 4:46–61. 10.7453/gahmj.2014.073 25694852 PMC4311559

[114] Kim AY Jang EH Choi KW Jeon HJ Byun S Sim JY. Skin conductance responses in major depressive disorder (Mdd) under mental arithmetic stress. PLoS ONE. (2019) 14:e0213140. 10.1371/journal.pone.0213140 30943195 PMC6447153

[115] Bourdillon N Jeanneret F Nilchian M Albertoni P Ha P Millet GP. Sleep deprivation deteriorates heart rate variability and photoplethysmography. Front Neurosci. (2021) 15:642548. 10.3389/fnins.2021.642548 33897355 PMC8060636

[116] Lo Martire V Caruso D Palagini L Zoccoli G Bastianini S. Stress & sleep: a relationship lasting a lifetime. Neurosci Biobehav R. (2020) 117:65–77. 10.1016/j.neubiorev.2019.08.024 31491473

[117] Göldi M Rasch B. Effects of targeted memory reactivation during sleep at home depend on sleep disturbances and habituation. Npj Sci Learn. (2019) 4:2. 10.1038/s41539-019-0044-2 31069114 PMC6497651

[118] Kushnir J Marom S Mazar M Sadeh A Hermesh H. The link between social anxiety disorder, treatment outcome, and sleep difficulties among patients receiving cognitive behavioral group therapy. Sleep Med. (2014) 15:515–21. 10.1016/j.sleep.2014.01.012 24767722

[119] Zalta AK Dowd S Rosenfield D Smits JAJ Otto MW Simon NM. Sleep quality predicts treatment outcome in Cbt for social anxiety disorder. Depress Anxiety. (2013) 30:1114–20. 10.1002/da.22170 24038728 PMC4043139

