# Erratum: Targeted memory reactivation during REM sleep in patients with social anxiety disorder

**DOI:** 10.3389/fpsyt.2023.1182122

**Published:** 2023-03-22

**Authors:** Frontiers Production Office

**Affiliations:** Frontiers Media SA, Lausanne, Switzerland

**Keywords:** sleep, REM sleep, dreaming, social anxiety, targeted memory reactivation, exposure therapy

An omission to the funding section of the original article was made in error. The following sentence has been added: “Open access funding was provided by the University of Geneva.”

The original article has been updated.

